# Identification of women at risk for hereditary breast and ovarian cancer in a sample of 1000 Slovenian women: a comparison of guidelines

**DOI:** 10.1186/s12885-021-08400-8

**Published:** 2021-06-03

**Authors:** Urska Kotnik, Borut Peterlin, Luca Lovrecic

**Affiliations:** 1 Clinical Institute of Genomic Medicine, University Medical Centre Ljubljana, Slajmerjeva 4, 1000 Ljubljana, Slovenia; 2 Biotechnical Faculty, University of Ljubljana, Jamnikarjeva 101, 1000 Ljubljana, Slovenia

**Keywords:** BRCA, Breast cancer, Referral criteria, Clinical guidelines, Family history

## Abstract

**Background:**

An important number of breast and ovarian cancer cases is due to a strong genetic predisposition. The main tool for identifying individuals at risk is recognizing a suggestive family history of cancer. We present a prospective study on applying three selected clinical guidelines to a cohort of 1000 Slovenian women to determine the prevalence of at-risk women according to each of the guidelines and analyze the differences amongst the guidelines.

**Methods:**

Personal and family history of cancer was collected for 1000 Slovenian women. Guidelines by three organizations: National Comprehensive Cancer Network (NCCN), American College of Medical Genetics in cooperation with National Society of Genetic Counselors (ACMG/NSGC), and Society of Gynecologic Oncology (SGO) were applied to the cohort. The number of women identified, the characteristics of the high-risk population, and the agreement between the guidelines were explored.

**Results:**

NCCN guidelines identify 13.2% of women, ACMG/NSGC guidelines identify 7.1% of women, and SGO guidelines identify 7.0% of women from the Slovenian population, while 6.2% of women are identified by all three guidelines as having high-risk for hereditary breast and ovarian cancer.

**Conclusions:**

We identified 13.7% of women from the Slovenian population as being at an increased risk for breast and ovarian cancer based on their personal and family history of cancer using all of the guidelines. There are important differences between the guidelines. NCCN guidelines are the most inclusive, identifying nearly twice the amount of women as high-risk for hereditary breast and ovarian cancer as compared to the AGMG/NSCG and SGO guidelines in the Slovenian population.

**Supplementary Information:**

The online version contains supplementary material available at 10.1186/s12885-021-08400-8.

## Background

Family history of cancer is the most important risk factor for breast and gynecological cancer development after sex and age [1]. The prevalence of a family history of breast and ovarian cancer is high at above 25% in the general population [2–4]. A pathogenic variant in hereditary breast and ovarian cancer (HBOC) predisposing BRCA1 and BRCA2 genes is present in 3–5% of breast cancer cases and 10% of ovarian cancer cases [5]. As genetic predisposition represents a frequent etiological factor for the development of breast and gynecological cancers, genetic analysis plays an important role in cancer prevention, counseling about other cancer types, and genetic counseling to other family members. Clinical guidelines recommend that women with breast and ovarian cancers are referred to genetic counseling because the identification of a pathogenic variant presents an opportunity for a differential treatment, especially since a new family of drugs, the PARP inhibitors, have recently been approved and they have the greatest efficacy in women who carry a BRCA pathogenic variant [6].

However, it is less common to refer unaffected women to genetic counseling for gynecological cancers, even when they harbor a family history of cancer and genetic counseling and testing would provide important information for their cancer risk evaluation [7]. Identification of a pathogenic variant in asymptomatic women presents an opportunity to tailor appropriate monitoring and surveillance for breast and other cancers, in addition to offering prophylactic, risk-reducing interventions [8]. The process of genetic testing begins with a referral from a general practitioner or oncologist to a genetic counselor; therefore, patients strongly rely on physician identification and referral for genetic counseling. Studies revealed that referral of women at risk for familial breast and ovarian cancer to a genetic specialist presents a challenge for physicians, consequently many high-risk women are not identified [9, 10].

Since general population screening for BRCA pathogenic variants is currently not recommended due to low general population prevalence (1 in 300 to 500) [11], it is important to focus on the optimal selection of women with higher risk from the general population. Professional organizations have developed several clinical guidelines to determine whether referral for genetic counseling and testing for individual patients or family members is appropriate (NCCN, ACMG/NSGC, SGO, and others) [12–16]. These guidelines, updated irregularly, discuss the minimum criteria based on women’s personal and family history of cancer and identify individuals with an increased risk of hereditary cancer in the family to warrant genetic counseling and testing, such as the number of relatives affected, the number of cancers in the same person, and the patient’s age at diagnosis of cancer [17].

Clinical guideline’s specificity and sensitivity for selection of pathogenic variant carriers from women with a confirmed diagnosis of breast or gynecological cancers have been estimated in several studies [18–21]. There have, however, only been a few previous studies to estimate the number of women from the general population who fit the high-risk criteria using NCCN guidelines released in years 1999–2015 and estimated the numbers of the high-risk women identified were reported to be less than 1% (NCCN 1999) [17], 7.8% (NCCN 2006) [22], and 14.1% (NCCN 2012) [23]. Another study identified 20.8% of women from the general population as high-risk using NCCN 2004 guidelines; however, personal and family history of breast and ovarian cancer in the tested cohort was well above average [24]. A recent study assessed that the population prevalence of unaffected individuals meeting the NCCN 2015 guidelines is 11.6% [25]. NCCN guidelines have since been updated multiple times and more recent versions have not yet been tested in the general population. ACMG/NSGC and SGO guidelines analyzed in this article were not previously tested in the general population. Moreover, it has not yet been evaluated how differences between the guidelines are reflected in the numbers of identified women as having an increased risk for hereditary breast and ovarian cancer when applied to the general population of women.

We aimed at comparing clinical guidelines for identification of women at risk and referral to genetic counseling for hereditary breast and ovarian cancer issued by three professional organizations [13–15] that were recently endorsed by the medical community for women with gynecologic cancers [26]: NCCN (2.2021) [13], ACMG in cooperation with NSGC (2015) [14], and SGO (2015) [15]. Furthermore, we applied those three guidelines to 1000 Slovenian women from the general population to select women with high risk for hereditary breast and ovarian cancer in a prospective study.

## Methods

### Study population and data collection

An interview was conducted amongst a thousand women from the general population, patients of the Outpatient Clinic of the Division of Gynecology and Obstetrics, University Medical Centre Ljubljana, Slovenia, between January 2018 and September 2019. Inclusion criteria for our study was female sex, age above 18 years, and patient status of any of the outpatient specialist gynecologic clinics of the Division of Gynecology and Obstetrics, UMC Ljubljana for various gynecologic diseases (primary gynecologic outpatient clinic, urodynamic outpatient clinic, internal medicine, sterilization, physiotherapy, and others). Exclusion criteria was the inability to communicate in the Slovenian language.

The women completed an interview that included: contact information, personal history of gynecologic and other cancers, previous genetic testing/pathogenic variant found in the family, and family history of gynecologic and other cancers. Information was collected on all known cancerous diseases within a family, age at diagnosis of cancer, family relation to the interviewee, type of cancer, and the bloodline of the relative. The questionnaire used to conduct these interviews was developed for this study (Additional file 1). The 1000 women interviewed represent 0.1% of all women in Slovenia. All patients gave informed consent for participation per the Declaration of Helsinki. The study was approved by the Slovenian National Medical Ethics Committee (0120–113/2018/4).

### Guidelines selection

Based on a recent endorsement by the medical community for women affected with gynecological cancers [26] we have selected the following three guidelines for comparison and analysis: 1) NCCN Clinical Practice Guidelines in Oncology: Genetic/Familial High-Risk Assessment: Breast, Ovarian and Pancreatic (Version 2.2021) [13], 2) ACMG in cooperation with NSGC: A practice guideline from the American College of Medical Genetics and Genomics and the National Society of Genetic Counselors: referral indications for cancer predisposition assessment (2015) [14], and 3) SGO: Society of gynecologic oncology statement on risk assessment for inherited gynecologic cancer predispositions. (2015) [15]. ‘Testing criteria’ were extracted from the NCCN guidelines and ‘referral criteria’ were collected from the ACMG/NSGC and SGO guidelines. Referral criteria are meant for identification of women at risk and their referral to a genetic specialist and testing criteria were developed for identification of women at risk and to be used as an indication for genetic testing [13–15]. Both of these criteria were developed for identification of individuals at risk for HBOC. NCCN guidelines state that an individual that fulfills the testing criteria should receive risk assessment, counseling and genetic testing [13]. ACMG/NSGC guidelines recommend that if the referral criteria are met, the affected individual should be referred to genetic consultation and genetic testing if indicated and available, as assessed by a genetic specialist [14]. SGO guidelines state, that all women who meet the referral criteria, should receive genetic counseling and be offered genetic testing [15].

### Statistical analysis

The agreement among guidelines was calculated using the kappa statistic of the interrater agreement, using the level of agreement for healthcare studies (0.40–0.59 weak, 0.60–0.79 moderate, 0.8–0.9 strong) [27].

## Results

### Characteristics of the study population

Our study population consisted of a group of 1000 women, aged 18 to 88 years old. The median age is 36 years old. Women presented both personal and family history of cancer. Namely, 3% of women had a personal history of breast and/or ovarian cancer. Family history of breast and/or ovarian cancer (considering first and second-degree relatives (FDR and SDR, respectively)) was reported in 21.1% of women in our cohort, and of those, 6.3% of women had a FDR with breast cancer and 1.7% of women had a FDR with ovarian cancer. No personal or family history of any cancer was reported by 27.8% of interviewees (Table 1).

**Table 1:** Characteristics of the study population

**Age**	% of women in our study
18–30	23.8
31–45	49.1
46–60	16.8
61+	9.4
NA	0.9
**History of cancer**	% of women in our study
Personal and family history of breast and/or ovarian cancer	1.1
Personal history of breast and/or ovarian cancer only	1.9
Family history of breast and/or ovarian cancer only	20.0
FDR with breast cancer	6.3
FDR with ovarian cancer	1.7
No personal or family history of any cancer	27.8

Legend: *FDR* first-degree relative, *NA* not available

### Application of guidelines

The three sets of guidelines for the identification and referral of women to genetic counseling have been applied to our study group. Details of criteria, characteristic for each of the three guidelines (degree of relation of relative with cancer, number of cases of cancer, age at diagnosis or type of cancer), and the number and percentage of women from our study population qualifying for each criterion with each guideline are shown in Table 2.

**Table 2:** Application of guidelines to women from the general population

Criteria indicating the important family history of HBOC	NCCN		ACMG/NSGC		SGO	
	Details of criteria		Details of criteria		Details of criteria	
	N	%	N	%	N	%
An individual at any age with a known pathogenic/likely pathogenic variant in a cancer susceptibility gene	Pathogenic variant in a cancer susceptibility gene present in the family		Pathogenic variant in a cancer susceptibility gene present in the family		*BRCA1* or *BRCA2* pathogenic variant present in the family	
	10	1	10	1	10	1
Two breast cancer primaries in a single individual	Present in patient/FDR/SDR, one at 46–50 years		Present in patient/FDR		Present in patient/FDR/ several SDRs/TDRs, one of them < 50	
	0	0	2	0.2	0	0
Two individuals with breast cancer on the same side of the family with at least one diagnosed before or at 50 years	Present in patient/FDR/SDR		/		One in patient/FDR and another in FDR/SDR/TDR	
	13	1.3	0	0	13	1.3
Ovarian cancer (including peritoneal and fallopian tube cancer)	Present in patient/FDR/ SDR		Present in patient /FDR		Present in patient /FDR/several SDRs/TDRs	
	57	5.7	29	2.9	29	2.9
Male breast cancer	Present in FDR/SDR		Present in FDR		Present in FDR/SDR/TDR	
	1	0.1	1	0.1	1	0.1
Breast cancer diagnosed before or at 45/50 years	Cancer diagnosed ≤45 years in patient/FDR/SDR		Cancer diagnosed ≤50 years in patient/FDR		Cancer diagnosed ≤45 years in patient/FDR/several SDRs/TDRs	
	44	4.4	35	3.5	24	2.4
Pancreatic or metastatic prostate cancer	Pancreatic cancer in patient/FDR or breast cancer in patient/FDR/SDR and pancreatic cancer in SDR/TDR or high risk prostate cancer in patient/FDR or any prostate cancer in FDR/SDR and FDR/SDR/TDR with ovarian/pancreatic/high risk prostate cancer or patient/FDR/SDR with breast cancer and FDR/SDR/TDR with prostate cancer (either breast cancer at 46–50 or high risk prostate cancer)		/		Pancreatic cancer in patient/FDR and ≥ 2 FDR/SDR/TDR with breast/ovarian (peritoneal, fallopian tube)/ prostate cancer	
	35	3.5	0	0	0	0
An individual with a personal and/or family history of two cancers	Breast cancer in patient/FDR/SDR and FDR/SDR/TDR with ovarian (peritoneal, fallopian tube)		/		Breast cancer in patient/FDR and FDR/SDR/TDR with ovarian (peritoneal, fallopian tube) cancer	
	22	2.2	0	0	10	1.0
An individual with a personal and/or family history of three or more cancers	≥3 cases of breast/prostate cancer in patient/FDR/SDR/TDR		≥3 cases of breast/ovarian (peritoneal, fallopian tube)/pancreatic/aggressive prostate cancer in patient/FDR/SDR/TDR without cousins		Breast cancer in patient/FDR and ≥ 2 FDR/SDR/TDR with breast cancer in or breast cancer in patient/FDR and ≥ 2 FDR/SDR/TDR with pancreatic/aggressive prostate cancer	
	8	0.8	7	0.7	3	0.3
Number of women meeting the guidelines*	132	13.2	71	7.1	70	7

*Some of the women fit more than one criterion (the sum of criteria is larger than the number of women meeting the guidelines). Legend: < before the age; ≤ before or at the age; ≥2/≥3: 2 or more/ 3 or more; / - or; *FDR* first-degree relative: mother, sister, daughter, *SDR* second-degree relative: half-sister, grandmother, aunt, niece, granddaughter, *TDR* third-degree relative: cousin, great-grandmother, great aunt, great-granddaughter. All relatives must be in the same bloodline. Some of the women fit more than one criterion (the sum of criteria is larger than the number of women meeting the guidelines). High risk prostate cancer is defined as metastatic, intraductal or cribriform histology or high or very high risk group prostate cancer [13]

There are differences in the criteria definition and description in the NCCN, ACMG/NSGC, and SGO guidelines. Consequently, different guidelines identify a varied number of women as high-risk for hereditary breast and ovarian cancer (Table 2). NCCN guidelines identify 13.2% of women from our study population as high-risk. ACMG/NSGC and SGO guidelines are considerably less inclusive, identifying less than half of the women that were identified by NCCN, i.e., 7.1 and 7.0% of our study population, respectively.

**Fig. 1:**
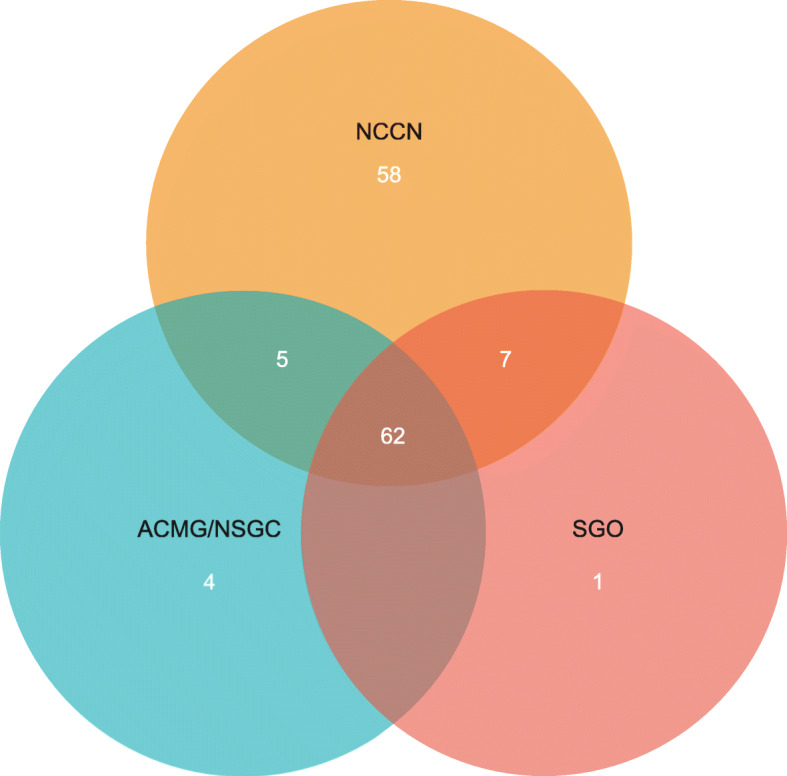
Number of women identified by each of the three guidelines

Our analysis showed that 23.0% of women from our cohort had a personal or family history of breast or ovarian cancer up to second-degree relatives. Out of these women, 60%, representing 13.7% of all women, were identified as high-risk for hereditary breast and ovarian cancer by at least one guideline; 6.2% of all women were identified as high risk for hereditary breast and ovarian cancer by all of the guidelines analyzed (Fig. 1). NCCN identified 58 women that were not identified as high-risk by the other two guidelines. ACMG/NSGC identified four women and SGO identified one woman that were only identified as high-risk by those guidelines. Five women were identified as high-risk by both NCCN and ACMG/NSGC guidelines, and seven women were identified as high-risk for HBOC by NCCN and SGO guidelines.

The most women were identified based on the criterion of ovarian cancer in the family (5.7% with NCCN, 2.9% with ACMG/NSGC and SGO), where there is no age limit defined and based on the criterion of early onset of breast cancer (4.4% with NCCN, 3.5% with ACMG/NSGC, and 2.4% with SGO). NCCN identifies an additional 3.5% of women as high-risk, based on the criterion of pancreatic/ prostate cancer in the family, however, more than half (2.1%) of those women do not meet any other criteria and/or are not identified as high-risk by the other two guidelines.

We used the Kappa statistic of the interrater agreement for quantification of the agreement between the guidelines. This test confirms that there is a strong agreement between the women identified as high-risk for hereditary breast and ovarian cancer syndrome between ACMG/NSGC and SGO guidelines with 0.87; 95% CI (0.81–0.93); *p* < 0.001, and only a moderate agreement between NCCN guidelines and the remaining two guidelines (Table 3).

**Table 3:** Agreement between guidelines

	ACMG/NSGC	SGO
**NCCN**	0.63; 95% CI (0.54–0.71); *p* < 0.001	0.65; 95% CI (0.57–0.73); *p* < 0.001
**SGO**	0.87; 95% CI (0.81–0.93); *p* < 0.001	–

Legend: *NCCN* National Comprehensive Cancer Network, *ACMG/NSGC*- American College of Medical Genetics/National Society of Genetic Counselors, *SGO* Society of Gynecologic Oncology

## Discussion

A comparison of the three referral guidelines has revealed differences among the criteria with each of the guidelines. The criteria an individual at any age with a known pathogenic/ likely pathogenic variant in a cancer susceptibility gene within the family and male breast cancer in family identify the same number women from our study group as high risk for breast and ovarian cancer with all three analyzed guidelines. Using other criteria, different guidelines identify a varied number of women as high risk for HBOC.

NCCN guidelines are the most inclusive amongst the guidelines, identifying nearly double the number of women compared to the other two guidelines. A group of 58 women have been identified as high-risk only by NCCN, with 21 of those women identified due to having a FDR/SDR with pancreatic or prostate cancer in the family. NCCN guidelines included this criterion in a 2019 update [12] based on the research that 2–5% of unselected adenocarcinoma patients harbor BRCA1/2 pathogenic variant [28], making BRCA1/2 pathogenic variants the most common genetic cause of pancreatic cancer [29] and so, an attractive candidate gene for genetic testing. Moreover, pancreatic cancer has a high mortality rate, and the possibility to test the affected relative is, therefore, time limited [30]. Prostate cancer is the second most common cancer in men [31] and has a high rate of heritability as well [32]. A study has shown a 1.2% prevalence rate of pathogenic BRCA1/2 variants in unselected prostate cancer patients [33]. Patients with metastatic prostate cancer are known to harbor pathogenic variants in a cancer predisposition genes, including BRCA1 and BRCA2, far more frequently than patients with localized prostate cancer [34], therefore genetic testing of all patients with metastatic prostate cancer may be beneficial, especially since screening and treatment options are available [29]. NCCN guidelines identify 5.7% of women as high risk based on their personal or family history of ovarian cancer. A genetic predisposition of ovarian cancer is frequent: previous studies have shown that 13–20% of unselected ovarian cancer patients carry a pathogenic BRCA1/2 variant [35, 36]. In line with this evidence, genetic testing is universally recommended for all ovarian cancer patients and their FDRs by the analyzed guidelines [13–15]. This is especially important, since screening and early detection of ovarian cancer is challenging [13]. NCCN additionally recommends genetic testing for women, who only had a SDR with ovarian cancer. In addition to those women, NCCN guideline is also the only guideline that identifies women with a SDR with any of the following as high-risk: breast cancer before age of 45 years, two breast cancer primaries or two SDRs with breast cancer (1 before 50 years) or ovarian (peritoneal/tubular) cancer. Those women with SDRs account for the remaining 37 women exclusively identified by NCCN. A previously published study revealed that including SDRs in cancer risk assessment is frequently beneficial, therefore, this criterion has important implications [37].

ACMG/NSGC and SGO guidelines identify 7.1 and 7.0% of women as high-risk, respectively, with some differences amongst the guidelines. The criterion that uniquely identifies women as high-risk with ACMG/NSGC guidelines is having a FDR with breast cancer at ages between 45 and 50 years present in the family. This criterion results in the identification of six additional women. Testing first-degree relatives of women with the diagnosis of breast cancer between ages 45 and 50 years might be reasonable, since a recent study showed that the peak incidence of breast cancer in BRCA1 pathogenic variant carriers occurred between the ages 41 and 50 [38]. SGO guidelines identify one woman that is not identified by the other two guidelines, since SGO are the only guidelines that consider two cases of breast cancer (one in a patient/FDR; one younger than 50 years old) in a family sufficient for the identification of high-risk women if one of the cases is third-degree relative (TDR). In our cohort, they additionally identify a woman that has a mother (FDR) and a maternal cousin (TDR) with breast cancer, where the cousin was younger than 50 years at diagnosis. Identification of only one additional woman in our group is supported by a previous study that has shown that information on the breast cancer history of cousins or other TDRs rarely improves the accuracy of risk assessment in a family, therefore, the additional effort of incorporating data on the cancer history of TDRs is seldom beneficial and the inclusion of this criterion in the identification process may have a low yield [39].

Application of various combinations of criteria to our study group reveals that 6.2% of women from our cohort were concordantly identified as high-risk by all guidelines. The criteria that selected these women as high-risk group were a BRCA1/2 pathogenic variant present in the family, patient or a FDR with two or more primary breast cancers, ovarian cancer, breast cancer before age 45 or male breast cancer, or had a combination of three or more specific cancers present in the family. These shared criteria may represent the core criteria of the guidelines, identifying the highest risk women, since it has already been suggested that agreement of multiple guidelines might be considered for the selection of women with the highest risk for the pathogenic variant, viewing each guideline as an ‘expert’ and all guidelines as an ‘expert panel’ [22, 23].

It has recently been observed that the prevalence of BRCA pathogenic variant carriers is higher than previously estimated [40] and that current clinical guidelines fail to identify an important proportion of patients carrying the pathogenic variants [18, 19, 21, 41]. Consequently, recent updates of the referral guidelines recommend referral of women with less remarkable family history of cancer [8]. Studies that examined older versions of NCCN guidelines for HBOC referral of women from the general population identified more women with each update [17, 22, 23]. Our study reveals that the NCCN 2.2021 guidelines identify 13.2% of women from the general Slovenian population as high risk for HBOC. This is an increase from the referral rates in most of the aforementioned studies [17, 22] and comparable to the referral rate of 14.1% in a recent study in Brazilian population [23] and might account for the expansion of the criteria in the recent NCCN guidelines.

The aim of the identification of high-risk women in the population is to reduce morbidity and mortality in those women by referring them to appropriate screening and prevention [42]. Our study identified 13.7% of women from the general population older than 18 years as being at an increased risk for HBOC based on their personal and family history of cancer. This subset of women represents a high-risk population with possible implications for cancer prevention, as recommended by the guidelines [42]. In the Slovenian population of 2 million, that means more than 130.000 women may need more frequent breast and ovarian cancer screening than women from the general population, which would present an important public health burden. This finding may support further considerations about healthcare implications in Slovenia.

The limitation of our study is that we cannot disclose the specificity and sensitivity of each of the guidelines for the identification of pathogenic variant carriers in high-penetrance cancer genes due to several reasons. Most importantly, the 1000 women from our study population have not been molecularly tested and their BRCA status has not been determined. Because of that, we cannot assess the sensitivity of the guidelines, since we are unable to determine whether some of our average risk population might be asymptomatic carriers of a pathogenic variant in a cancer predisposition gene without a family history of cancer and, therefore, part of the high-risk population. This can be partly explained by the fact that pathogenic variant carriers sometimes lack indicative family of cancer because of different reasons: imperfect reporting of cancer disease in the family, presence of adoption or risk-reducing surgeries in a family, families with few female relatives or a small family size [13]. Secondly, it is important to note that only a subset (20–30%) of familial breast cancer is explained by a pathogenic variant in BRCA1/2 or another highly penetrant cancer gene [43, 44]. Put differently, even when performed, a molecular analysis does not account for breast and ovarian cancer susceptibility in high-risk women with familial breast cancer without an identifiable pathogenic variant, making the specificity of the guidelines for the identification of high-risk population difficult to assess. Further research is needed to determine the rate of cancer predisposition in this high-risk population. Another limitation of our study is self-reporting of personal and family history of cancer diagnosis by the participants in the study without the ability to confirm the diagnoses by consulting the patient records, however, patient-reported data of family history of breast and ovarian cancer has been found reliable in a previous study [45].

## Conclusion

In conclusion, the analysis of cancer family history of 1000 women from the general population shows that NCCN, ACMG/NSGC, and SGO guidelines identify 13.7%, an important proportion of women as high-risk for hereditary breast and ovarian cancer. NCCN guidelines identify nearly double the number of women compared to the remaining two guidelines as having an increased risk for HBOC in the Slovenian general population.

## Supplementary Information

**Additional file 1.** Questionnaire used to conduct the interviews.

## Data Availability

The datasets used and/or analyzed during the current study are available from the corresponding author on reasonable request.

## Abbreviations

ACMG/NSGC: American College of Medical Genetics/National Society of Genetic Counselors
FDR/SDR/TDR: First/second/third-degree relative
HBOC: Hereditary breast and ovarian cancer
NCCN: National Comprehensive Cancer Network
SGO: Society of Gynecologic Oncology
