# Supplementary material for: Identification of women at risk for hereditary breast and ovarian cancer in a sample of 1000 Slovenian women: a comparison of guidelines
Source: BMC Cancer. 2021 Jun 3;21:665. doi: 10.1186/s12885-021-08400-8 (PMC8176743; doi:10.1186/s12885-021-08400-8)
Supplement: Supplementary file 1 — Additional file 1. Questionnaire used to conduct the interviews. [file 12885_2021_8400_MOESM1_ESM.docx]

Title of the study:

**IDENTIFICATION OF WOMEN AT RISK FOR HEREDITARY BREAST AND OVARIAN CANCER**

A questionnaire for women, patients of the outpatient clinic of the Division of Gynecology and Obstetrics, University Medical Centre Ljubljana

**CONTACT INFORMATION**

**Name and surname**

|  |
| --- |

**Address (street, city, and postcode)**

|  | |
| --- | --- |
|  |  |

**Telephone number**

|  |
| --- |

**Email address**

|  |
| --- |

**PERSONAL CHARACTERISTICS**

| Birth date |  |
| --- | --- |

**Has your doctor ever told you that you have any of the following diseases?**

|  | Yes | No | Age at diagnosis |
| --- | --- | --- | --- |
| Breast cancer |  |  |  |
| Ovarian cancer |  |  |  |
| Pancreatic cancer |  |  |  |
| Endometrial cancer |  |  |  |
| Colorectal cancer |  |  |  |
| Cancer genetic syndromes, such as: Peutz-Jeghers, Cowden, Li-Fraumeni, Lynch, hereditary colorectal cancer |  |  |  |
| Other cancer – Which? (in case of more, list all) | Which? |  |  |
|  |  |  |  |

| Have you previously received radiation therapy to the chest for treatment of cancerous disease? | Yes | No |
| --- | --- | --- |
| Have you previously heard of the possibility of genetic testing to determine an increased risk of cancer in an individual? | Yes | No |
| Have you ever talked to your doctor about the possibility of genetic testing? | Yes | No |
| Were you previously involved in genetic counselling or testing for familial cancer? | Yes | No |

**Are you aware of any family member with a potential pathogenic variant in BRCA gene or other cancer-related gene (blood relationship)?**

| No – nobody in my family has ever been tested | Yes – genetic test in a family member was positive | No – genetic test in a family member was negative |
| --- | --- | --- |

|  | **Your height** (cm) |  | **Your weight** (kg) |  |
| --- | --- | --- | --- | --- |

| **How old were you at the time of your first menstrual period?** | 7-11 | | 12-13 | | | 14 or more | | I don’t know | |
| --- | --- | --- | --- | --- | --- | --- | --- | --- | --- |
| **How old were you at birth of your first child?** | No children | <20 | | | 20-24 | | 25-29 | | 30 or more |
| **Have you ever had a breast biopsy?** | Yes | | | | | No | | | |
| **IF YES,** how many? | 1 | | | | | More than 1 | | | |
| **IF YES**, have you ever had a breast biopsy with atypical hyperplasia? | Yes | | | No | | | I don’t know | | |

**FAMILY DISEASES**

1. Indicate if any of your relatives have had any of the listed diseases. **List which relative, age at their diagnosis, and whether the relative is from maternal or paternal side of the family.**

*Relatives considered: children, siblings, parents, half siblings, grandparents, uncles, aunts, nieces/nephews, grandchildren, cousins, great-aunts/uncles, grandparents, great-grandchildren. Ignore non-blood relatives.*

|  | Breast cancer (mark if bilateral) | Ovarian cancer | Prostatic cancer (mark if metastatic, high risk) | Pancreatic cancer | Endometrial cancer | Colorectal cancer |
| --- | --- | --- | --- | --- | --- | --- |
| **Which relative (1)**: |  |  |  |  |  |  |
| **Age at diagnosis (1)**: |  |  |  |  |  |  |
| **Maternal (M) or Paternal (P) relative (1):** |  |  |  |  |  |  |

| **Which relative (2)**: |  |  |  |  |  |  |
| --- | --- | --- | --- | --- | --- | --- |
| **Age at diagnosis (2)**: |  |  |  |  |  |  |
| **Maternal (M) or Paternal (P) relative (2):** |  |  |  |  |  |  |

**An example:**

| **Which relative (3)**: |  |  |  |  |  | *grandmother* |
| --- | --- | --- | --- | --- | --- | --- |
| **Age at diagnosis (3)**: |  |  |  |  |  | *60* |
| **Maternal (M) or Paternal (P) relative (3):** |  |  |  |  |  | *M* |

1. **Consider any other cancers your relatives have had!**

Especially consider the following cancers:

Gastric cancer, ureteral cancer, bile duct cancer, small bowel cancer, kidney cancer, brain tumor, thyroid cancer, leukemia, skin cancers, lymphomas, lung cancer, testicular cancer, bone cancer, adrenal gland tumor, gastrointestinal polyps **or any other cancer**.

List your relatives and information on their diseases.

|  | **1** | **2** | **3** | **4** | **5** | **6** | **An example:** |
| --- | --- | --- | --- | --- | --- | --- | --- |
| **Which relative:** |  |  |  |  |  |  | *Cousin* |
| **Which cancer (list all):** |  |  |  |  |  |  | *Bone cancer Lung cancer* |
| **Age at diagnosis** |  |  |  |  |  |  | *48*  *51* |
| **Maternal (M) or Paternal (P) relative:** |  |  |  |  |  |  | *P* |
